# Evaluation Of Congestion Levels in Septic Patients Admitted to Critical Care Units with a Combined Venous Excess-Lung Ultrasound Score (VExLUS) – a Research Protocol

**DOI:** 10.24908/pocus.v8i1.16188

**Published:** 2023-04-26

**Authors:** Miguel Romano, Eduardo Viana, José Diogo Martins, Rogério Corga Da Silva

**Affiliations:** 1 School of Medicine, Minho University Braga Portugal; 2 Internal Medicine, Unidade Local de Saúde do Alto Minho Viana do Castelo Portugal; 3 Intensive Care Unit, Unidade Local de Saúde do Alto Minho Viana do Castelo Portugal

**Keywords:** POCUS, VExUS, lung ultrasound, Sepsis, Septic Shock, Venous congestion

## Abstract

Sepsis is defined as a life-threatening organ dysfunction caused by a dysregulated host response to infection with a high mortality rate. Septic shock is a subset of sepsis with manifest circulatory dysfunction (use of vasopressors and persistent elevation of lactic acid) . As stated in literature, in addition to the use of empiric antibiotics and control of the infectious focus, intravenous fluid therapy is an essential intervention to promote hemodynamic stabilization. However, the literature also describes harmful outcomes related to fluid overload. Hemodynamic management in critically ill patients has traditionally focused on maintaining adequate cardiac output and arterial blood pressure by relying on fluid administration and/or vasopressor/inotropic support. However, organ perfusion is affected by other important factors, such as venous pressure, which can be overlooked. The evaluation of lung congestion with point of care ultrasound (POCUS), as a signal of extravascular fluid, and, more recently, a venous excess Doppler ultrasound (VExUS) grading system, are parameters for the assessment of the fluid status of the patient and organ congestion. Our main hypothesis is that adding a modified lung ultrasound score to the VExUS protocol could provide higher sensitivity and earlier identification of fluid overload, guiding the clinician in the decision of fluid administration in patients with sepsis.

## Background

Sepsis is defined as a life-threatening organ dysfunction caused by a dysregulated host response to infection which is associated with an in-hospital mortality greater than 10%. On the other hand, septic shock should be defined as a subset of sepsis in which particularly profound circulatory, cellular, and metabolic abnormalities are associated with a greater risk of mortality (hospital mortality rates greater than 40%) 1. Currently, in addition to the use of empiric antibiotics and control of the infectious focus, intravenous fluid therapy is an essential intervention to promote hemodynamic stabilization. However, observational studies describe worse outcomes related to fluid overload and increased mortality^2^, ^3^ and greater use of fluid-related medical interventions such as thoracentesis, paracentesis, hemodialysis, and use of diuretics 4. As stated in the FENICE study, routine fluid response prediction is rarely used, with safety limits easily surpassed 5. Considering this, it is necessary to find an accurate method to assess the congestive status of these patients and promote an early recognition of fluid overload.

Hemodynamic management in critically ill patients has traditionally focused on maintaining adequate cardiac output and arterial blood pressure by relying on fluid administration and/or vasopressor/inotropic support 6, 7. However, organ perfusion is affected by other important factors, such as venous congestion, which can be overlooked as an hemodynamic parameter, and could be of critical importance 8, 9. 

Point of care ultrasound (POCUS) at the bedside allows the clinician to assess intravascular and pulmonary fluid overload. The correlation of lung POCUS and extravascular fluid is a well-known parameter for the assessment of the global fluid status of the patient 3. Misinterpretation in POCUS is a risk. Initially, with the generalization of the POCUS use in critical patients, physicians misleadingly relied on static inferior vena cava (IVC) sole measurement as an assessment of fluid responsiveness 10, 11. 

Similarly, it is essential to measure and interpret central venous pressure (CVP) with care and criteria. When utilized as a guide or trigger for fluid resuscitation, misunderstandings are common. A rise in CVP may not be accompanied by a corresponding rise in cardiac output (CO), as De Backer et al. skillfully point out in their review, and without measuring this variable (CO), a rise in CVP solely represents an increase in venous return and an increase in preload 12, 13. This is insufficient to predict the patients response to fluid administration. 

Recently, Beaubien-Souligny et al. proposed a venous excess Doppler ultrasound (VExUS) grading system to quantify organ congestion 14. This protocol evaluates the amount of venous congestion in the abdominal organs, specifically by scanning the portal, hepatic, and intrarenal veins. Doppler interrogation of these vessels yields a specific pattern corresponding to a level of venous congestion: normal, mild, or severe. Although VExUS grading is a novel concept, the role of these individual Doppler waveforms in the assessment of right atrial pressure has been previously established 15.

**Aims and Hypothesis**

Our main hypothesis is that by assessing septic patients with a noninvasive ultrasound protocol, adding a modified lung ultrasound score to the VExUS protocol could provide greater sensitivity and earlier identification of fluid overload to help clinicians decide whether to administer supplemental fluid therapy or suspend its administration. The key secondary aims are to: i) evaluate the possible relation between the different VExLUS grades and the adverse effects of intensive fluid administration, namely diuretic use, the need of thoracocentesis/paracentesis, need for renal replacement therapy; ii) Identify the possible relation between a congestive findings of renal ultrasound and new or worsening acute kidney injury and iii) identify the possible association between higher VExLU score and length of stay and mortality. 

## Methods/Design

### Study design

The combined VEXUS and Lung Ultrasound Score (VExLUS) for septic patients in critical care units, is a single-centered, observational, prospective study conducted at Unidade Local de Saúde do Alto Minho, Viana do Castelo, Portugal.

### Study settings and population

Participation in the study is proposed to patients admitted in the intensive care unit or intermediate care unit of Unidade Local de Saúde do Alto Minho with the diagnosis of sepsis or septic shock. Patients are screened, and if all inclusion criteria are met, without the presence of any exclusion criteria, patient written information is given, and non-opposition consent is collected. With all these criteria fulfilled, patients are enrolled in the study. 

**Figure 1:**
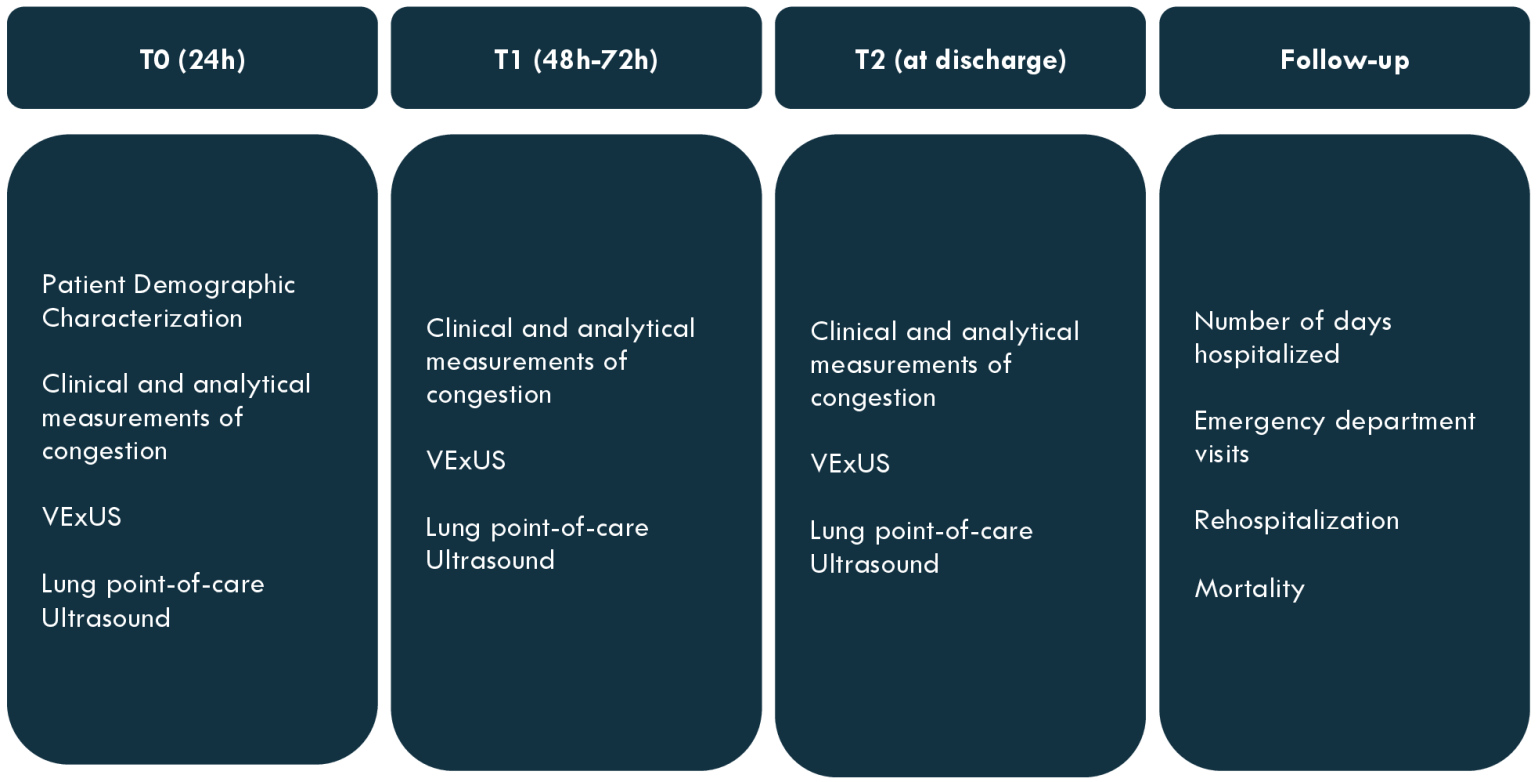
Moments of examination and obtained variables.

The proposed protocol is divided into three moments of examination as presented in Figure 1:

- T0: in the first 24 hours after admission
- T1: within 48-72 h after admission
- T2: at the moment of discharge

**Figure 2:**
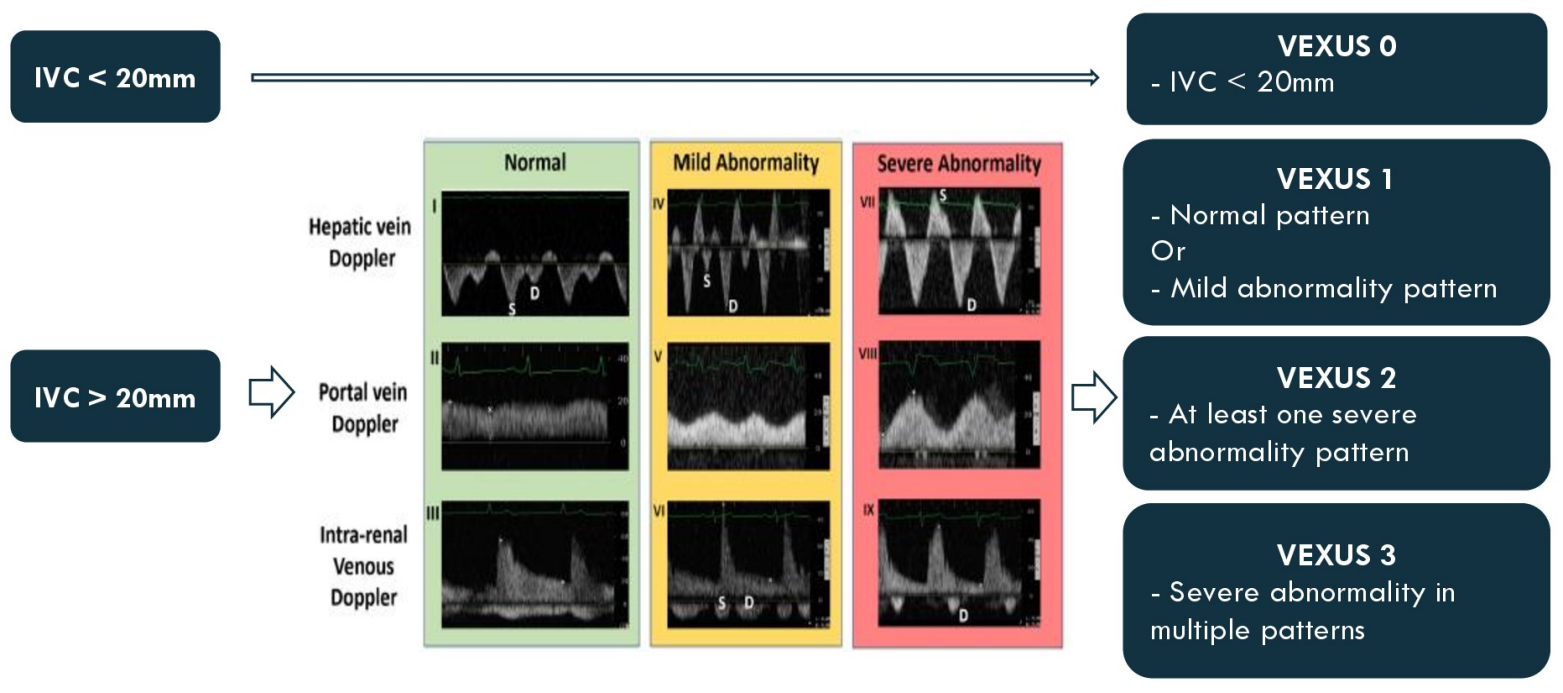
VExUS classification.

**Figure 3:**
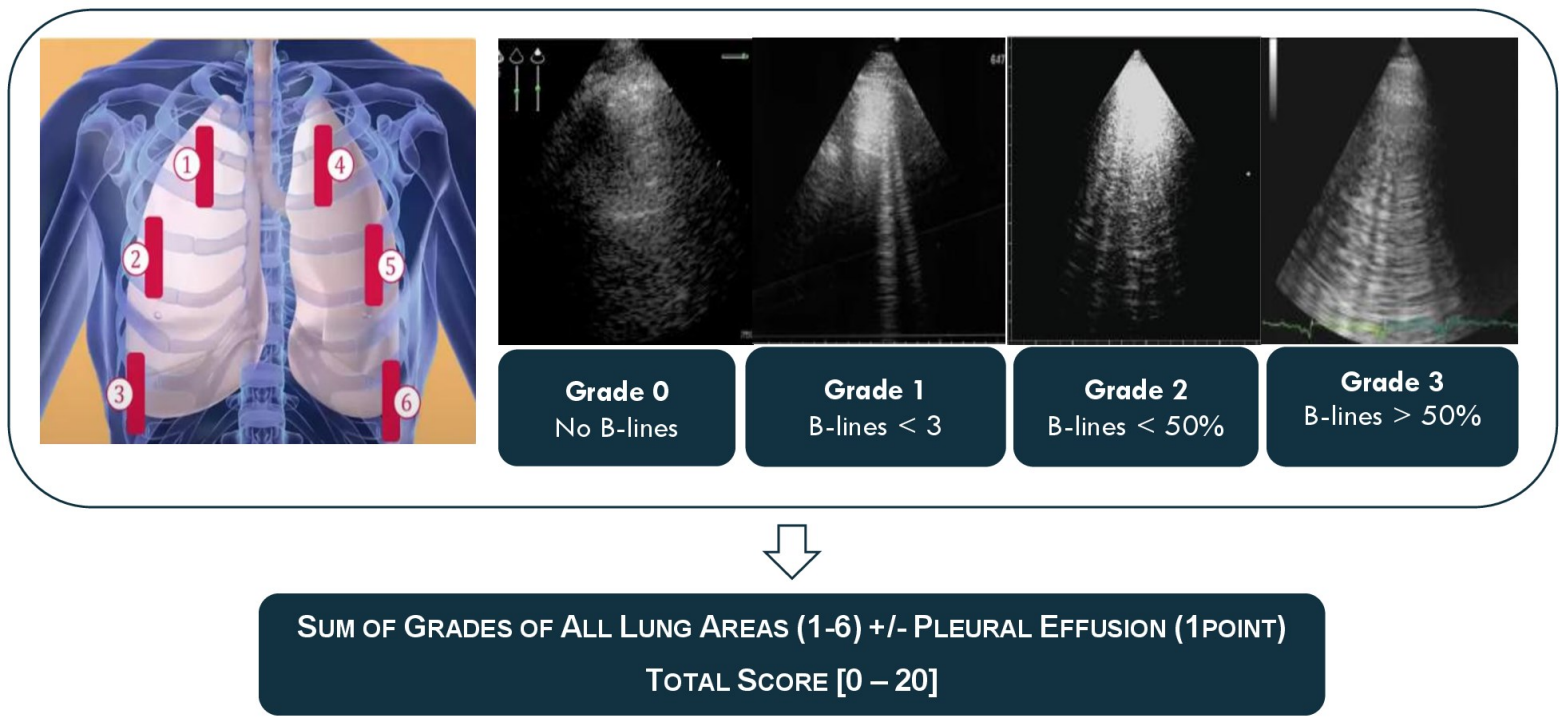
Modified Lung Ultrasound Score.

In each of these moments, a systematic ultrasound exam is made. First, the VExUS score is calculated as presented in Figure 2. Then, a systematic lung ultrasonography exam is performed where 6 areas are evaluated and each is classified from 0 to 3 as explained below in Figure 3.

### Inclusion Criteria

For this study, adult patients (≥ 18 years old) with the diagnosis of sepsis or septic shock and clinical indication to be admitted in critical care units are recruited. Sepsis should be suspected to be the primary cause of their acute illness by the treating physician, consistent with the Surviving Sepsis Campaign Guidelines (2021) 16:

- Life-threatening organ dysfunction
- Diagnosis of infection

Some patients diagnosed with sepsis will not meet the criteria, but will be eligible for the study if the treating physician makes a clinical diagnosis of severe sepsis or septic shock. 

### Exclusion Criteria

1. Refusal to participate
2. Patients in hemodialysis program
3. Patients with previously known conditions that interfere with portal Doppler assessments, namely liver cirrhosis, or severe tricuspid regurgitation with structural heart disease. If any of these conditions are identified during the present episode, the patient would also be excluded.
4. Age < 18 years
5. Patients subjected to withdrawal of care
6. Hemodynamic instability due to active hemorrhage
7. Acute coronary syndrome
8. Indication for immediate surgery
9. Received CPR within 24 hours of enrollment
10. Pregnancy

### Characterization of the participants

Data regarding each patient will be collected in the 3 different moments mentioned above (T0, T1 and T2). At moment T0 will take place the initial demographic and clinical characterization of the participant:

- Age
- Gender
- Weight, height, and BMI
- Frailty Score
- Comorbidities (Charlson Comorbidity Index)
- Symptoms at presentation and sepsis starting point
- Clinical and analytical values will be collected in the 3 moments (T0, T1, T2):
  - Presence/Absence of
  - Heart failure
  - Renal Failure
- Hydroelectrolytic disturbances
- SOFA score (total and discriminated)
- Central venous pressure
- Cumulative fluid balance
- Cumulative diuretic dosage
- Rohde score
- Need and days of vasopressor
- Class of antibiotics
- Fluid related intervations
- Laboratory values: lactate, reactive C protein, procalcitonin, BNP, creatinine, urea, sodium, potassium, bilirubin (total and direct), complete hemogram

After discharge (T2) and until the end of the study, the follow-up phase, other variables will be collected:

- Number of hospitalization days
- Hospital readmission (< 30 days)
- Emergency department readmission
- Mortality

### Point of care ultrasonography (POCUS)

POCUS is performed by trained practitioners with ultrasound scanners using the following parameters: low frequency (2–5 MHz) transductors, convex (abdominal transductors) or phased array (cardiac transductors) type probes. 

### Venous excess Doppler ultrasound (VExUS) grading system

The VExUS grading system is calculated following the steps of the original paper. First the IVC is measured and, if bigger than 20 mm, the portal, hepatic and intrarenal veins are scanned. Doppler interrogation of these vessels yields a specific pattern corresponding to a level of venous congestion: normal, mild, or severe. According to the results, the VExUS score is obtained as follows: VEXUS 0: IVC < 20mm; VEXUS 1: IVC ≥ 20mm with normal patterns or mild abnormalities; VEXUS 2: IVC ≥ 20mm with severe abnormality in at least one pattern and VEXUS 3: IVC ≥ 20mm and severe abnormalities in multiple patterns. 

Considering the technical difficulties in achieving the best ultrasound images for this protocol, the authors allow the use of the best window and view consider by the operator.

### Lung point of care ultrasonography

Lung ultrasound is performed in 6 different areas as shown in Figure 3. Assuming that in a critical care unit, patients will be mainly in dorsal decubitus, the patient will be evaluated in 3 anterior areas in the right hemithorax and other 3 in the left. Each area contemplates only one intercostal space. Four ultrasound aeration grades are defined: Grade 0: A lines; Grade 1: less than 3 B lines; Grade 2: less than 50% of B lines and Grade 3: more than 50% of B lines. Each of the 6 lung areas is examined, and the final aeration score, ranging from 0 to 18 points, is the sum of each regional ultrasound score. We also determine the presence of pleural effusion for each hemithorax adding one more point to the final score (for each side). 

### VexLUS Score

The final score is obtained as follows:

1. Each grade of VeXUS is transformed into a points-score:
  1. Grade 0 = 0 points
  2. Grade 1 = 5 points
  3. Grade 2 = 10 points
  4. Grade 3 = 15 points
2. Modified Lung Ultrasound Score ranges from 0 to 20 points
3. Both scores are added and classified:
  1. No congestion: 0 to 10 points
  2. Slight congestion: 11 to 20 points
  3. Moderate congestion: 21 to 30 points
  4. Severe Congestion: 31 to 35 points

### Trial Outcomes

#### Primary Outcome

- VExLUS score as an early marker for global fluid overload

#### Secondary Outcome

- Correlation of VExLUS score with cumulative fluid balance
- VExLUS score for prediction of adverse effects of intensive fluid administration (namely, acute kidney injury, respiratory failure, the need of diuretic use to correct overt fluid overload)
- Isolated kidney venous congestion as an initial marker of fluid excess
- Incidence of acute kidney injury
- Incidence of congestive respiratory failure (not attributed to other causes)
- Incidence of hidroelectrolytic disturbances
- Impact of VExLUS score in mortality
- Impact of VExLUS score in ICU length of stay

### Study Timeline

The study is projected to have a duration of 2 years. Preliminary results analysis will be performed after the first 6 months and 1 year after the beginning.

### Data collection, management, and analysis

#### Data collection and management

All data related to this study are collected using a standardized electronic case report form (eCRF) and based on valid documents (patient medical record). The confidentiality of patients and their personal health information is always maintained by restricting access to patient records and eCRF.

It is important to notice that the responsible physicians for the examined patient will not have access to the collected data, ensuring that no therapeutic measures will be done considering the POCUS results.

#### Statistical analysis

The collected data will be analyzed using Statistical Package for the Social Sciences (SPSS) ®. The statistical processing will be made using the mentioned program. Many different data sets are described in this study. Quantitative data are described using means and SD. Qualitative data are described using numbers, percentages and 95% CIs. P value < 0.05 will be considered of statistical significance.

## Ethics Considerations

### Legal obligations and approval 

Clinical research standards were safeguarded, in accordance with the recommendations contained in the Declaration of Helsinki, applicable national and international laws, namely Law No. 21/2014 and Directive No. 2001/20/EC, of the European Parliament and of the Council, of 4 April, Good Clinical Practice (ICH-GCP) and General Data Protection Regulation EU 2016/679, in force since 25 May 2018.

### Ethical Principles

This protocol was submitted for approval by the ethics committee of the Alto Minho Local Health Unit. The dignity, integrity, self-determination, privacy, and confidentiality of the personal information of the participating subjects were protected by the research team of the proposed study. The POCUS performed at the patient's bedside was performed in accordance with the best recommended practices, being complementary to the physical examination.

The collected data will be stored in a database and will be pseudo-anonymized from the moment of inclusion in it. Thus, the analysis of these will not allow the identification of the patient. Pseudo-anonymization: First name initial and last name initial, followed by the month and year of birth (example: Rogério Esteves Domingues Corga da Silva, 24.09.1984. It would be registered as: “RE091984”).

Participants will be informed of the researchers' objectives, methods, funding sources, possible conflicts of interest and institutional affiliations. All participants must sign an informed consent and authorize the study and. In case of critical illness and/or inability to sign, the authorization may be provided by the next of kin. Nevertheless, all participants have the right to refuse participation in the study or withdraw their consent at any time without the need for justification. 

## Trial status

Inclusions started on October 2022.

## Limitations

The technical expertise required to administer the VEXLUS protocol might limit its adoption by clinicians. This is aggravated by the fact that EKG will not be used for the interpretation of hepatic vein Doppler.

## Conflicts of Interest

The authors have no conflicts of interest to declare. 
